# Beyond a century of discovery: the global and persistent burden of underdiagnosis in von Willebrand disease

**DOI:** 10.1016/j.rpth.2026.103359

**Published:** 2026-01-20

**Authors:** Omid Seidizadeh, Rezan Abdul-Kadir, Pier Mannuccio Mannucci, Flora Peyvandi

**Affiliations:** 1 Fondazione IRCCS Ca’Granda Ospedale Maggiore Policlinico, Angelo Bianchi Bonomi Hemophilia and Thrombosis Center, Milan, Italy; 2 Department of Obstetrics and Gynaecology, Royal Free Hospital NHS Trust, London, UK; 3 Katharine Dormandy Haemophilia and Thrombosis Centre, London, UK; 4 Institute for Women’s Health, University College London, London, UK; 5 Università degli Studi di Milano, Department of Pathophysiology and Transplantation, Milan, Italy

**Keywords:** global health, underdiagnosis, von Willebrand disease, VWD, women’s health

## Abstract

In February 2026, von Willebrand disease (VWD) will mark a century since its first description by Dr Erik Adolf von Willebrand. VWD is the most common inherited bleeding disorder and characterized predominantly by mucocutaneous bleeding. Despite remarkable advances in understanding its biology, diagnostic assays, genetics, and treatment, VWD remains widely underdiagnosed and misdiagnosed. Population-based studies estimate a prevalence between 0.8% and 1.6%, with 1 in 1000 individuals carry clinically significant VWD phenotypes, but global registry-reported prevalence averages only 25.6 per million, highlighting a striking gap between expected and identified cases. Underdiagnosis is driven by low awareness among health care providers, clinical and laboratory heterogeneity, assay variability, limited access to specialized testing, and misclassification as other bleeding disorders. Although VWD affects both sexes equally, women and girls are disproportionately impacted, with up to 90% experiencing heavy menstrual bleeding, 30% to 50% facing postpartum hemorrhage, and many missing school or workdays due to bleeding. Median diagnostic delay in women can exceed 14 years, often with multiple severe bleeding episodes prior to recognition. Disparities are particularly pronounced in low- and middle-income countries, where only severe cases are typically identified. Addressing these gaps requires global harmonization of diagnostic standards, increased awareness among health care providers, broader use of bleeding assessment tools, expanded laboratory capacity, and integration of sex-specific and precision medicine approaches. Coordinated policy, education, and awareness initiatives are essential to ensure early detection, equitable care, and optimal outcomes. The goal for the second century of VWD is that all patients are accurately diagnosed and appropriately treated.

## 1 Introduction

In February 2026, a century will elapse since the first description of von Willebrand disease (VWD). The story began in April 1924, when a 5-year-old girl from Föglö, one of the Åland Islands in the Gulf of Bothnia between Finland and Sweden, was admitted to hospital with severe, recurrent bleeding from nose and gums. Her case prompted a thorough family investigation by Dr Erik Adolf von Willebrand, an internist at the Deaconess Hospital in Helsinki, Finland. The girl’s parents were cousins, and a familial bleeding tendency was evident: among 11 siblings, several had experienced similar symptoms, and 3 had died from gastrointestinal hemorrhage [1,2]. The proband girl died at the age of 14 years, during her fourth menstruation from massive uterine bleeding. Nearly 2 years later, in February 1926, Dr von Willebrand published his seminal report describing this previously unrecognized bleeding disorder under the name hereditary pseudohaemophilia, now called after him congenital VWD [1,2]. In contrast to hemophilia as a classical hereditary bleeding disorder, this condition affected both sexes equally and was predominantly characterized by mucocutaneous bleeding rather than by soft tissue and joint bleeding [3].

VWD is globally recognized as the most common inherited bleeding disorder and is characterized by substantial phenotypic and diagnostic complexity. Over the past century, remarkable advances have transformed the understanding of the disease, from the identification of the causative glycoprotein, von Willebrand factor (VWF), to the development of sophisticated diagnostic assays, *VWF* gene cloning and detailed genetic characterization, as well as major innovations in treatment [3]. Despite a century of scientific progress, underdiagnosis and misdiagnosis remain main global challenges.

This condition is caused by quantitative or qualitative defects in the VWF glycoprotein. The most common phenotypic manifestations include epistaxis, easy bruising, mucocutaneous bleeding, heavy menstrual bleeding (HMB), postpartum hemorrhage, and bleeding after surgery. Less frequent presentations include gastrointestinal bleeding, hematomas, and hemarthroses [[3], [4], [5]]. VWD is classified into 6 major types: quantitative deficiencies underline type 1 (partial VWF deficiency) and type 3 (complete VWF deficiency), while qualitative defects cause type 2, including types 2A, 2B, 2M, and 2N [[3], [4], [5]].

In this Viewpoint, we discuss the striking gap between the expected prevalence of VWD and diagnosed cases, the global burden and consequences of underdiagnosis, the reasons why underdiagnosis persists after a century, and ultimately propose strategies to guide the second 100 years of VWD care and research.

## 2 Expected Global Prevalence of VWD vs Registered Patients

Estimating the actual prevalence of VWD, particularly for milder forms such as type 1 and some type 2 VWD cases, is complicated by the heterogeneity of clinical and laboratory manifestations, variable penetrance and expressivity, and ambiguous diagnostic criteria. Despite these challenges, numerous studies using clinical, laboratory, and genetic approaches have attempted to define the global burden of VWD.

Data from several national population-based studies have yielded prevalence estimates ranging from 0.82% in Italy [6] to 1.6% in the United States [7], with intermediate values reported in Arabic and Turkish populations (1.23%) [8], mixed US cohorts (1.3%) [9], and Saudi Arabia (1.5%) [10]. A Canadian study further suggested that the prevalence of symptomatic VWD is at least 1 in 1000 in primary care settings [11], implying that >8 million people worldwide should exhibit clinically significant manifestations of the disease. In striking contrast, registry-based analyses report substantially lower figures [12]. Using data from the 2022 World Federation of Hemophilia Annual Global Survey, national registries in Australia, Canada, and the United Kingdom, as well as from the published literature, the mean observed global prevalence of VWD was estimated at 25.6 ± 48.8 cases per million people [12]. Importantly, prevalence was strongly associated with the level of national income: high-income countries reported 60.3 cases per million, compared with 12.6, 2.5, and 1.1 cases per million in upper-middle–, lower-middle–, and low-income countries. The condition was also more frequently reported among women than that in men in registries [12]. Indeed, VWD is more commonly diagnosed in females than that in males owing to such women-specific physiological events as menstruation and childbirth [3,5].

Recent large-scale genetic epidemiology studies have provided further insights, suggesting that the true prevalence of VWD, when defined by known *VWF* pathogenic variant frequencies, may be considerably higher than registry data indicate [13,14]. Estimated per 1000 individuals, prevalence was 11 for type 1, 1.3 for type 2A, 1.7 for type 2B, and 1.5 for type 2M, with type 2N (31 per million) and type 3 (1.2 per million) occurring much rarely [14]. These estimates translate to a global burden exceeding several million individuals genetically affected by causative *VWF* variants.

**Figure 1:**
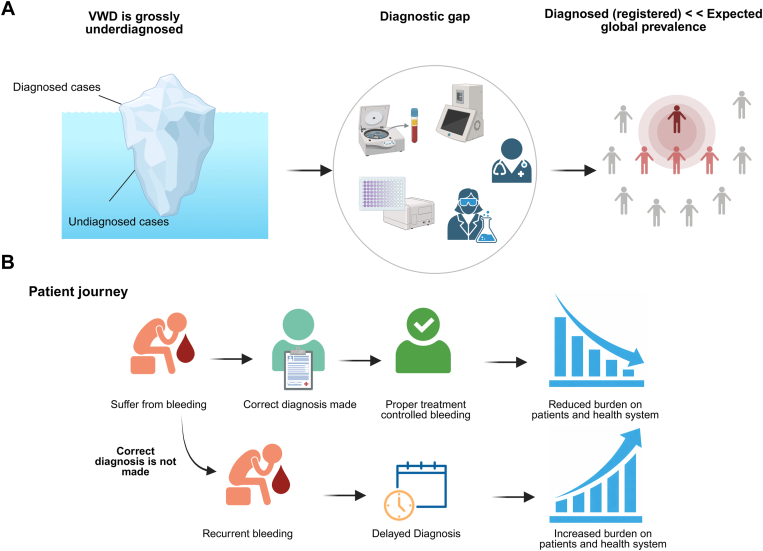
Global underdiagnosis of von Willebrand disease (VWD). Population-based studies estimate a prevalence of 0.8% to 1.6% with at least 1 in 1000 individuals carry clinically significant VWD phenotypes. However, registry-reported prevalence averages only 25.6 per million, highlighting a major gap between expected and diagnosed cases. This gap is more pronounced in low- and middle-income countries, where the rate of diagnosed VWD cases is substantially lower (often <1 case per million), and only severe phenotypes are typically identified. Created with BioRender.com.

As noted earlier, symptomatic VWD is estimated to affect at least 8 million individuals globally, yet according to the most recent World Federation of Hemophilia Annual Global Survey (1999-2024), only 115,672 cases were registered, representing only approximately 1.4% of the expected numbers [15]. In the United States, computer modeling based on insurance claims suggests that between 35,000 and 387,000 people may have symptomatic but undiagnosed VWD or related bleeding disorders, highlighting that the vast majority of individuals with VWD remain unidentified and undertreated [16]. Taken together, these diverse lines of evidence underscore that VWD is not only the most prevalent inherited bleeding disorder but also among the most complex and persistently underdiagnosed (Figure 1A).

## 3 The Global Burden of VWD Underdiagnosis and Its Consequences

The underdiagnosis and misdiagnosis of VWD imposes substantial morbidity upon patients, reduces their quality of life, and increases health care burden worldwide (Figure 1B). Registered data reveal large international disparities in diagnosis, which are heavily influenced by national income levels and health care infrastructure. For example, registration rates (2018/2019 World Federation of Hemophilia Annual Global Survey) are the lowest in South Asia (0.6 per million population) and highest in Europe and Central Asia (50.9 per million), both far below the symptomatically expected prevalence of approximately 0.1% (1 in 1000) [17]. In low- and middle-income regions, for several reasons (discussed later), underrecognition predominantly affects mild and moderate cases, with only the most severe cases (type 3 VWD) typically being identified [17].

**Figure 2:**
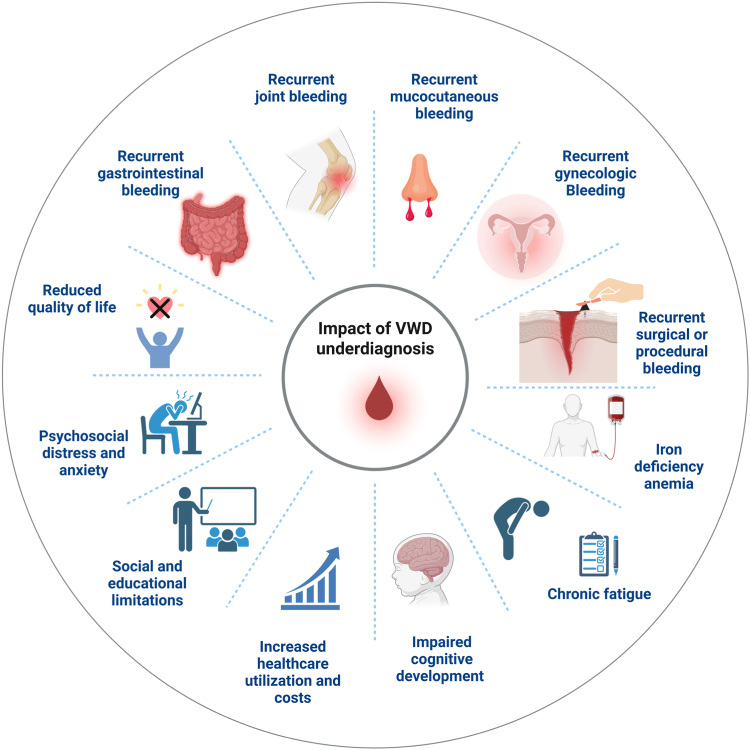
Clinical and health system consequences of undiagnosed or misdiagnosed von Willebrand disease (VWD). Delayed or missed diagnosis leads to significant clinical, psychosocial, and economic burden on patients and health care systems. Created with BioRender.com.

The clinical and psychosocial consequences of delayed or missed diagnosis are profound (Figure 2) [[18], [19], [20], [21], [22], [23], [24], [25], [26], [27], [28]]. Patients are exposed to untreated bleeding, which may result in life-threatening events during miscarriage, childbirth, or surgery, as well as joint or gastrointestinal hemorrhages, recurrent epistaxis, easy bruising, bleeding in mucosal tracts, and trauma- or procedure-related bleeding. These manifestations often lead to secondary complications, including iron deficiency anemia, chronic fatigue, and increased health care utilization. Quality of life is impaired, particularly for children and for women/girls (see below), many of whom experience HMB, social limitations, and anxiety. For example, iron deficiency can impair cognitive development in children and affect their quality of life.

Altogether, considering that VWD is grossly underdiagnosed, even in high-income countries, many patients experience the disease without receiving a correct diagnosis and appropriate treatment, creating a substantial global burden on patients, families, society, and health care systems.

## 4 Special Attention to Women and Girls with VWD

Women and girls with VWD experience a disproportionately high disease burden due to their inherited hemostatic impairment combined with gynecologic and obstetric challenges [29]. HMB is the most common symptom, affecting 60% to 90% of women, often leading to chronic iron deficiency anemia, fatigue, and reduced quality of life [5,30]. Postpartum hemorrhage occurs in approximately 30% to 50% of affected women, several-fold higher than in the general population [5,30,31]. Cultural and social factors can further exacerbate this burden: HMB is frequently stigmatized, thus delaying recognition and diagnosis, and sometimes resulting in unnecessary hysterectomies. Although some obstetric and gynecologic professional societies recommend screening for VWD prior to hysterectomy for abnormal uterine bleeding, data suggest that this occurs in <1% of cases; implementing these guidelines more broadly will be critical to avoiding unnecessary surgery and achieving timely diagnosis [32]. Recurrent or severe bleeding also causes educational and occupational impairment, with many girls and women missing days of school or work each month (Figure 2) [33,34]. Regarding sexuality, women with VWD experience greater sexual restriction than men and report a markedly higher frequency of postcoital bleeding than that in the general population. Sexual restrictions are more commonly reported by premenopausal than by nonmenstruating women, primarily due to HMB [35].

Diagnostic delays are common and have serious consequences. In a study, the median time from first symptom to diagnosis was 14.2 years; 40% of women had experienced 3 or more significant bleeding events, and 41% had at least 1 severe episode requiring emergency care or transfusion before diagnosis [18]. Even when HMB is treated, nearly 50% of women continue to experience this complication, and almost all report limitations in daily activities due to menstruation [24]. Women and girls with VWD also report greater menstrual pain than the general population, and women with HMB may score lower on cognitive assessments than those without [23,36]. Reproductive challenges are notable, with miscarriage rates ranging from 3% to 29%, and rates of anemia and iron deficiency (including during pregnancy) between 4.2% and 22.5% [36].

Taken together, these findings underscore the substantial physical, cognitive, and psychosocial burden of VWD particularly in women and girls, particularly in low- and middle-income countries, and highlighting the urgent need for early recognition, careful monitoring, and multidisciplinary management strategies.

## 5 Reasons for Underdiagnosis and Misdiagnosis

**Figure 3:**
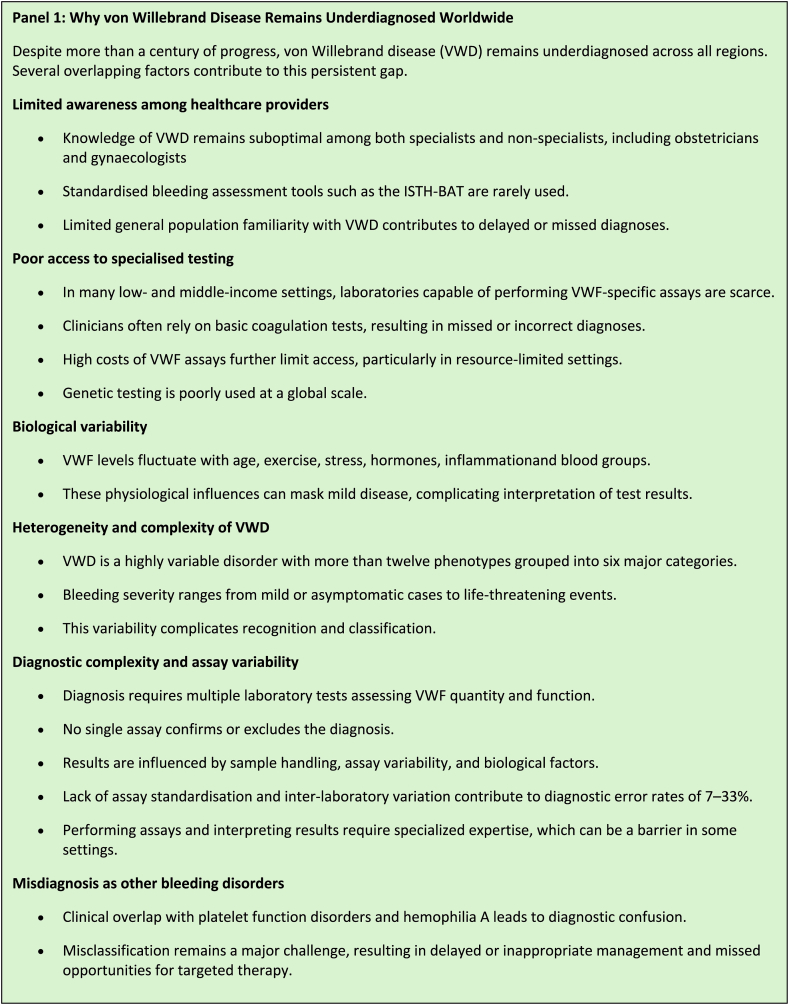
Why von Willebrand Disease Remains Underdiagnosed Worldwide.

A question that is looming large is why, even after >100 years since Dr von Willebrand first description of the disease and notwithstanding so many advances in diagnosis and understanding of its pathophysiology, VWD remains underdiagnosed globally. Several reasons should be considered for this paradox (Figure 3).

### 5.1 Limited awareness

Awareness of VWD remains limited among both health care professionals, particularly among nonhematologists, and in the general public, leading to delayed or missed diagnoses. The disease’s name, being less familiar and less iconic than hemophilia, may further reduce suspicion and recognition in clinical practice. Awareness is often particularly low among general practitioners, obstetricians, and gynecologists, even though VWD is a well-recognized underlying cause for HMB and other bleeding symptoms in women [30]. HMB, often the presenting symptom for women with mild-moderate VWD, itself remains underdiagnosed and undertreated due to normalization of bleeding during menstruation, social stigma, lack of standardized assessment tools and systematic approach to investigation and management as well as gender inequality in access to health care [37]. A recent survey among physicians and researchers in thrombosis and hemostasis showed that knowledge of VWD treatment options and diagnostic approaches was suboptimal (only 65% and 35%), highlighting a major gap in disease awareness even among expert clinicians [38]. An additional study showed a general lack of awareness and education pertaining to bleeding disorders among nonhematologists (internal medicine residency training) [39]. Standardized bleeding scores such as the International Society on Thrombosis and Haemostasis (ISTH) Bleeding Assessment Tool (BAT) are underused in clinical practice, limiting the early recognition of abnormal bleeding and contributing to delayed or missed diagnosis of VWD [40].

### 5.2 Poor access to specialized testing

In many regions, particularly low- and middle-income countries, access to VWF assays, including both basic and specialized laboratory testing, is absent or scarce [[41], [42], [43], [44]]. Without the highly specialized VWF tools that are required for proper VWD diagnosis and typing, clinicians often rely on basic coagulation tests, which are insufficient to identify VWD or accurately classify its subtypes. Consequently, many patients remain undiagnosed or misdiagnosed and inadequately treated [42,43]. High costs of VWF assays further limit access, particularly in resource-limited settings [42,43].

### 5.3 Biological heterogeneity

VWF levels are highly variable and influenced by multiple physiological and genetic factors, contributing to the complexity of VWD diagnosis. Plasma VWF is an acute-phase reactant that fluctuates throughout an individual’s lifespan in response to age, stress, exercise, infection, hormonal changes, and inflammation [3,40,45]. Accordingly, VWF measurements obtained during these conditions in individuals suspected of having VWD may yield falsely normal results, potentially leading to missed or delayed diagnosis [40]. Additionally, individuals with blood group O naturally have lower baseline VWF levels, which can mimic or mask the presence of VWD [40]. This intrinsic variability, combined with differences in bleeding phenotypes and incomplete penetrance, makes it challenging to reliably identify affected individuals and to distinguish mild VWD from normal physiological variation.

### 5.4 Heterogeneity and phenotypic complexity

VWD is a remarkably complex and heterogeneous disorder, encompassing >12 distinct phenotypes that the current guidelines classify in 6 major groups [4]. Its clinical spectrum ranges from mild or even asymptomatic cases to severe, life-threatening bleeding episodes, as seen in the original Finnish family described by Dr Erik Adolf von Willebrand. This wide phenotypic variability continues to pose major diagnostic challenges and contributes significantly to the underrecognition of the disease.

### 5.5 Diagnostic complexity and assay variability

Diagnosing VWD is inherently challenging due to the complex interplay between clinical symptoms and laboratory measurements. Multiple assays (ie, >10 different assays) are required to assess VWF quantity and functions, such as VWF antigen, platelet-dependent VWF activity assays, collagen-binding assays, factor VIII coagulant activity, VWF multimer analysis, ristocetin-induced platelet agglutination assay and VWF binding to factor (F)VIII assay (VWF:FVIIIB) [3,46,47]. No single assay can confirm or reject VWD diagnosis, and most assays have inherent limitations, variable sensitivity, and interlaboratory differences, which can lead to inconsistent results, and sometimes misclassification of VWD types. Moreover, significant preanalytical (eg, sample handling), analytical and postanalytical issues, timing of collection, and concurrent illnesses can further affect the assay diagnostic accuracy. Furthermore, several assays are not standardized yet [46]. This diagnostic complexity contributes significantly to both underdiagnosis and misdiagnosis, particularly in settings without access to standardized or specialized laboratory testing. Lack of appropriate expertise in performing and interpreting the results of VWF assays is an additional reason for the underdiagnosis of VWD [46,47].

Laboratory assay variability and incomplete evaluation of VWF function further increase the risk of misclassification. A study evaluating the quality of testing in North American laboratories reported diagnostic error rates of approximately 10% for type 1 VWD and 33% for type 2 VWD samples [48]. In contrast, analyses from the Royal College of Pathologists of Australasia Quality Assurance Program demonstrated lower error rates of 7.5% and 14.3% for type 1 and type 2 VWD samples [41].

### 5.6 Misdiagnosis as other bleeding disorders

The clinical overlap between VWD and other inherited or acquired bleeding disorders can lead to misdiagnosis. Mild VWD, in particular, can present with mucocutaneous bleeding symptoms resembling those of platelet function disorders, while quantitative factor FVIII deficiencies (eg, in type 3 and type 2N) may mimic mild hemophilia A. Although comprehensive data are lacking, existing studies indicate that misclassification or misdiagnosis of VWD remains a recognized challenge in clinical practice [46,49,50].

## 6 The Way Forward—The Second 100 Years

**Figure 4:**
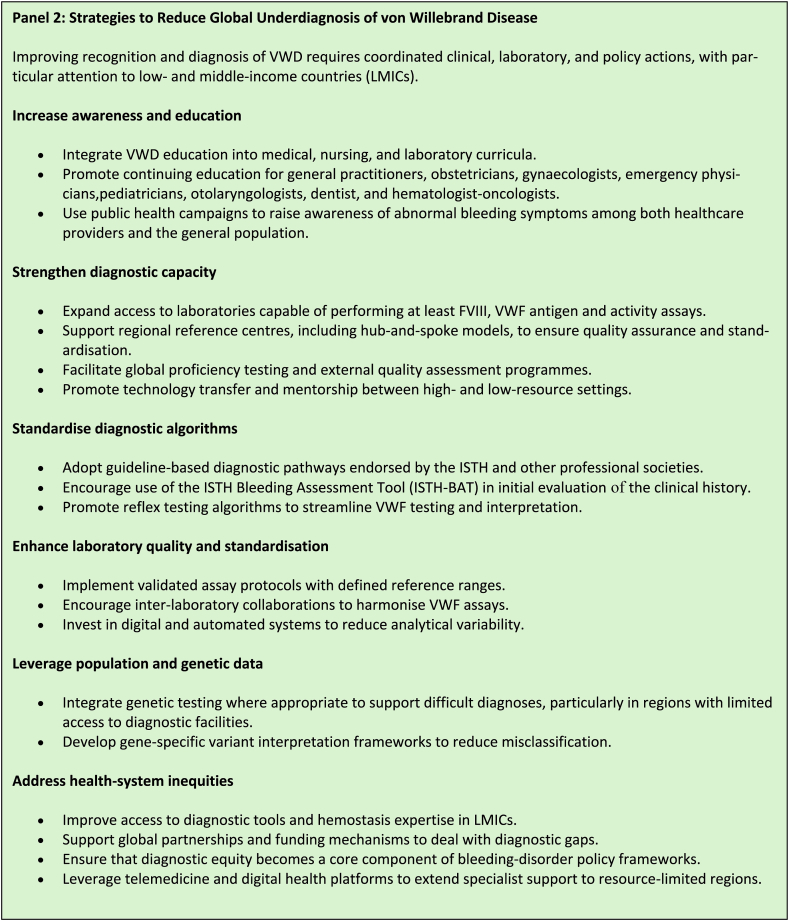
Strategies to Reduce Global Underdiagnosis of von Willebrand Disease.

Looking ahead, advancing the diagnosis and management of VWD will require coordinated global action. Harmonization of diagnostic standards and broader implementation of BATs are essential to ensure early and accurate recognition (Figure 4). Building capacity through targeted training programs for both health care providers and the general population, establishment of regional and reference laboratories (based upon hub-and-spoke models), and development of genetic databases representing diverse populations will strengthen diagnostic precision. Expanding the access to laboratories capable of performing at least FVIII, VWF antigen and activity assays in order to establish the initial diagnosis of VWD should be prioritized. Special emphasis should be placed on low- and middle-income countries, where limited resources, lack of trained personnel, and restricted access to specialized diagnostics contribute to more widespread underdiagnosis [[42], [43], [44]]. Ensuring affordable access to essential reagents and therapies, promoting technology transfer through international partnerships, and establishing mentorship networks between high- and low-resource settings are crucial to improving equity in care delivery (Figure 4). Advocacy efforts should aim to integrate VWD into the global noncommunicable disease and maternal health agendas, raising awareness of its broader public health impact. Leveraging telemedicine, digital health platforms, and surveillance systems may help to bridge diagnostic gaps in resource-limited regions. Effective communication and collaboration between clinicians, laboratory personnel, and researchers remain critical to optimize patient care and improve outcomes.

Addressing underdiagnosis will also require systematic strategies, with the goal of standardizing diagnostic algorithms, expanding access to specialized laboratory testing, and incorporating population-specific genetic and phenotypic data. Together, these measures can ensure timely diagnosis, appropriate management, and equitable care for all individuals with VWD, laying the foundation progress for the next century.

## 7 Conclusion

A century after the discovery of VWD, remarkable progress has been made in understanding its pathophysiology, clinical and genetic spectrum, and management [3,51]. However, underdiagnosis and misdiagnosis remain widespread, reflecting persistent gaps in awareness, diagnostic capacity, and access to care worldwide. As we enter the second century of VWD, it is imperative to increase awareness, prioritize equity, promote early and accurate diagnosis through standardized, easy-to-use BATs, and harness precision medicine, including accessible VWF assays, genetic testing, and individualized therapies. These measures are essential to optimize outcomes for all patients. Looking forward, the goal for the second century of VWD is clear: to ensure that virtually all individuals affected by this condition are properly diagnosed and receive appropriate and timely treatment.
